# Diagnosing Cochlear “Dead” Regions and Its Importance in the Auditory Rehabilitation Process

**DOI:** 10.1016/S1808-8694(15)30109-9

**Published:** 2015-10-19

**Authors:** Cristiane Padilha, Michele Vargas Garcia, Maristela Julio Costa

**Affiliations:** a Specialist, Audiologist.; b Specialist, Audiologist.; c PhD. Audiologist. Universidade Federal de Santa Maria-UFSM.

**Keywords:** hearing, audiologic diagnosis, cochlear dead regions

## Abstract

A good audiologic diagnosis is increasingly more important in the practice of audiology, in order to understand patients’ needs for selection and fitting of hearing aid devices.

**Aim:**

Show recent literature that mention the concept of cochlear dead regions, diagnostic strategies and its relevance in the process of selection and fitting of hearing aids.

**Methods:**

to carry out a bibliographical survey on dead cochlear regions. Dead cochlear regions were described as regions where inner hair cells and/or adjacent neurons do not work. Therefore, in these regions, the information generated by basilar membrane vibration is not transmitted to the central nervous system. However, a tone at a frequency correspondent to that of dead regions, provided it being sufficiently intense, can be perceived in regions near this zone where inner hair cells and/or nervous fibers still work.

**Conclusion:**

The identification of dead regions in the cochlea is used to obtain better results in the process of selection and fitting of hearing aids because the pieces of information generated by inner hair cells to the auditory nerve are important to better identify sounds, mainly those related to speech.

## INTRODUCTION

It is extremely important for the professional who works with audiology to have theoretical and practical knowledge in order to carry out a proper audiological diagnosis. It is based on the knowledge of how much and how the patient hears that one may think of the patient’s need for hearing aids.

Increasingly today, patients with hearing impairment are looking for auditory rehabilitation, and a great part of its success is related to the quality of auditory diagnosis, because hearing aid calibration is directly related to the information acquired in the exam.

Individuals with sensorineural hearing loss may present different types of cochlear failures, and there may be lesions in the outer hair cells (OHC) and/or inner hair cells (IHC), and the type of lesion impacts the individual’s response when they hear sound stimuli.

Moore et al. (1999a)^1^ reported that hearing loss by injury to the outer hair cells can not be higher than 50 dB in the low frequencies and 65dB in the high frequencies. Any hearing loss above the values stated above point to the involvement of inner hair cells, and in these cases there is injury to both, the outer and inner hair cells.

According to Zemlin (2002)^2^ the OHC are responsible for 5% of the information transmitted to the auditory nerve, while the IHC account for 95% of this transmission.

Thinking about the differences in the results found in hearing aid fitting in individuals with sensorineural hearing loss, however with different audiometric configuration (flat or in slope) is that Moore and Glasberg (1997)^3^ proposed the study of the cochlear functioning and the responses of external and internal hair cells when we hear sound stimuli. Based on this study, the authors described the so called cochlear dead zones, regions were the inner hair cells and/or adjacent neurons are not functional. Thus, in these regions, the information generated by the vibration of the basilar membrane is not transmitted to the central nervous system. Nonetheless, a sound in a frequency corresponding to the dead zone, if intense enough, may be perceived in regions near this zone, where the inner hair cells and/or nerve fibers are still functional.

Moore (2001a)^4^ reported that hearing thresholds above 75-80 dB in low frequencies and above 90 dB in the high frequencies indicate a probable presence of dead zones in the cochlea.

Cochlear dead zones can not be diagnosed based on the audiogram. Thus, Moore (2000)^5^ created a test called TEN (Threshold Equalizing Noise) in order to make this diagnosis. Other diagnostic means, reported by other authors, are also described.

Identifying cochlear dead zones has helped in selecting and fitting hearing aids, because the information generated by the IHC to the auditory nerve is important for a better sound recognition, especially speech sounds.

With these cochlear dead zones, the device’s amplification region shall be prescribed with caution, so that the patient may have the best possible hearing aid performance in relation to speech recognition, since the IHC are inactive.

Considering that studies on the cochlear dead zone have only recently started, and that this diagnosis is yet to be inserted in the clinical routine of audiology, the goal of the present investigation is to discuss recently published literature involving concepts of cochlear dead zones, diagnostic strategies and its importance in the selection and fitting of hearing aids.

## LITERATURE REVIEW

### Cochlear Dead Zone Theory

According to Moore and Glasberg (1997)^3^, cochlear dead zones are regions where the inner hair cells and/or adjacent neurons are not functional. Thus, in these regions, the information generated by the basilar membrane vibration is not transmitted to the central nervous system. Nonetheless, a sound in a dead zone frequency, if intense enough, may be detected in places with functional neurons and inner hair cells, through apical or basal spread of the vibration pattern. This causes a difficulty in decoding the acoustic information and even information overload in one same region.

Moore (2004)^6^ reassessed anatomical and psychophysiological fundaments of the dead zones theory. Initially, he defined “dead zone” as a cochlear region with significant reduction of the functions performed by inner hair cells and /or adjacent neurons, in such a way that the vibration generated in this region starts being detected by another, more functional one. Therefore, this region could be considered functionally dead and, very likely, little useful information to speech recognition would be transferred to that place.

### Diagnostic Strategies

Moore et al.(2000)^5^ proposed a clinical test to outline the cochlear dead zone areas using the TEN - Threshold Equalizing Noise. TEN was produced in order to equally mask a broad range of frequencies, from 125 to 15000 Hz. The level of the TEN noise is expressed in ERB (Equivalent Rectangular Bandwidth) referring to the bandwidth of the auditory filter; calibrated in such a way that the ERB level would be equivalent to the sound pressure level of hearing threshold, for example: the level of 70 dB/ERB usually masks the 70 dB SPL threshold. TEN noise and the sine test signal were recorded in independent channels in a CD-compact disc, in order to assess the different frequencies: 250, 500, 1000, 1500, 2000, 3000, 4000, 5000, 6000, 8000, 10000 Hz. Levels of sine signal and noise were presented through TDH50 phones and controlled in a two channel manual audiometer. Levels for noise presentation must be of 30, 50, 70 dB/ERB. In order to obtain the results, the control group was formed by 22 normal-hearing people, where the values of the masked thresholds in sound pressure level (dB SPL) were approximately equal to the values in TEM masking ERB level. The variation presented in this group was small in all frequencies assessed. A difference of 2 to 3 dB between masked thresholds and the absolute thresholds is considered normal, in other words, negative for the presence of dead zones in the cochlear.

Another group for study was formed by individuals with hearing loss of varied audiometric configurations. Of the 20 ears assessed, we identified 14 ears with dead zones in their cochleas. The cochlear dead zones were identified when the masked thresholds were at least 10 dB above absolute thresholds and 10dB above TEN ERB masking level. These results were confirmed through the Psychophysical Measure test of tuning curves. Presenting a signal at a given frequency, the curve peak was shifted from the dead zone to a neighboring functional area in the cochlea. The TEN test was considered by the authors as a simple and effective method to detect and outline cochlear dead zones. However, they stress that some aspects have to be considered in order to confirm the presence of dead zones in the cochlea, since most of the individuals in the study presented acquired hearing loss, commonly associated with hair cell lesions, and this did not happen in congenital hearing loss, therefore, interpreting the findings must be carried out separately for each case.

Moore (2004)^7^ suggested a new version of the TEN test for clinical practice, called TEN (AN), where all the intensity levels are specified in dB HL and not in dB SPL, facilitating the comparison between conventional audiometry absolute thresholds and masked thresholds with the test application. However, for these thresholds to be established in a more accurate way, 2 dB intervals (and not the 5 dB, used in the clinical routine) would be indicated. As to the range of frequencies, the new recommendations indicate test application between 500 and4000 Hz and masking intensity in one noise level only, from 85 to 90 dB/ERB. The TEN test criteria were developed based on its application in a relatively small group of adults with moderate to severe sensorineural hearing loss. These criteria may not be adequate for other populations and individual cases. The detection of a sound in the presence of noise depends on the signal/noise ratio for the basilar membrane site where it is detected and the individual’s hearing loss, which is partially associated with central mechanisms. Efficacy tends to reduce as age increase, elderly individuals need a signal/noise ratio 2 to 3 dB higher than what is necessary for younger individuals.

According to Moore and Alcántara (2001)^8^, one of the most direct psychophysical actions in frequency selectivity is the psychophysical tuning curve (PTC). The subject must detect a test signal in order for the PTC to be attained. The test signal has a fixed frequency and intensity in the presence of narrow band noise with variable central frequency. It is determined, for each one of the central frequencies the noise level necessary for the signal to become audible. Masking effectiveness is higher when its central frequency is near that of the test signal. The tip of the tuning curve (frequency at which the masking level is lower) always matches the test signal frequency in normal hearing individuals.

Different cochlear disorders have relations with changes in the tuning curve shape. When the inner hair cells are damaged, the neuronal fibers corresponding to this region present high thresholds both at the tip and at the curve end, however its configuration is not altered. When the damage happens only to the outer hair cells, the fibers that innervate this region present considerable sensitivity loss at its ends. The result of these changes is a broad “U”-shaped tuning curve, without a defined tip. In other cases, different levels of involvement may affect both the outer and inner hair cells, resulting in sensitivity and tuning losses.

The same authors describe the psychophysical test of tuning curves, used to identify dead cochlear zones. The methodology suggests the presentation of a sine signal of fixed frequency and at the level of 8 to 22 dB NS, with concurrent masking on the same side. The noise used was of narrow band, with variable center frequency and with masking band of 80Hz. The tuning curve was obtained by determining the necessary masking level to mask the sine signal for 12 varied frequencies, and the tuning frequency was identified as the frequency (point) obtained with the lowest masking level (more effective masking level). In normal hearing individuals, the tuning curve presented its peak at the tuning frequency equivalent to the frequency of sine signal presented, however, it was obtained using higher masking levels than the ones used for normal hearing people (Moore et al., 2000)^5^. The authors assessed five individuals with sensorineural hearing loss and varied audiometric patterns. The tuning curves’ peaks in the tested ears presented a shift towards the higher or lower frequencies when compared to the sine signal frequency, thus, all five presented results corroborating the presence of cochlear dead zones.

In order to diagnose cochlear dead zones, some researchers proposed the possibility of using other wide band noises, different from TEN.

Eguti (2002)^9^ evaluated 30 individuals with acquired hearing loss, sensorineural or mixed, determining absolute hearing thresholds, and thresholds under masking with white noise. The noise calibration was carried out through biological calibration in order to be effective. For biological calibration, the author counted on a sample with 10 normal hearing individuals, from both genders, in the age range between 21 and 38 years. She carried out tonal audiometry through air pathway in both ears of 10 individuals, and established absolute hearing thresholds by air pathway in the frequencies of 250 through 8000 Hz, in the following sequence: 1000, 1500, 2000, 3000, 4000, 6000, 8000, 750, 500 and 250Hz. Hearing thresholds were retested with the white noise being presented concurrently and ipsilaterally to the sound, in three different hearing levels: 30, 50, 70 dB HL. The noise must be increased until it masks the pure tone. The amount of masking necessary to mask the pure tone will be considered as the minimal level of effective masking. In this study, the different levels of noise proposed in the studies of Moore et al. (2000)^5^, Moore (2001a)^4^, were used, however, one may chose to run the test with the noise at 70 dB HL only - Moore et al. (2000)^5^. Thus, the test is shorter, less tiresome for the individual being tested and yields the same effect on the results.

In the sample of 10 individuals with normal hearing, there was a 5 dB HL variation over the absolute hearing threshold with the white noise, however, it was not considered as a positive result for the possible presence of dead zones in the cochlea, although Moore et al. (2000)^5^ found a 2 to 3 dB variation and standardized these values as the expected result for the lack of dead zones in the cochlea.

White noise has masking effectiveness in the frequency range between 750 and 6000 Hz, and less efficacy in the lowest and highest frequencies, that is, 250, 500 and 8000 Hz. According to Sanders and Hall III (2001)^10^, this may accrue from the fact that human hearing sensitivity is lower for lower frequencies and the response of white noise frequency drops in the frequency of 6000Hz. The acoustic influence of the audiometer probe (TDH49 ear phones) on the white noise, also generates less effectiveness in the ends of the frequency range, that is, 250, 500 and 8000Hz. The author concluded that the masking technique with white noise was reliable to identify the possible presence of dead zones in the cochlea.

The importance of diagnosing cochlear dead zones in the hearing rehabilitation process

Stelmachowicz (2001)^11^ said that high sound frequencies have a relevant role in speech and language development and, therefore, amplifying these frequencies should be carried out without restrictions, unless proven that amplifying higher frequencies may deteriorate speech information or that cochlear dead zones are identified.

Baer, Moore and Kluk (2002)^12^, considering the limited benefits presented to individuals with hearing losses above 55 dB in high frequencies and amplifying these frequencies, assessed speech recognition (SRI) in the presence of noise in ten individuals with hearing loss in high frequencies, five without it and five with cochlear dead zones. They started below 2000Hz and extended it from the threshold, all the way to the highest frequency assessed (10000 Hz). Before deciding on which amplification way was the most proper, the individuals were submitted to the TEN test in order to identify the presence of dead zones in the cochlea and outline how far it went. Speech and noise stimulus were amplified, and following that, submitted to the low-pass filter. In the SRT study, under broad band amplification situation (up to 7500 Hz) and filtered speech (low pass at 2000 Hz), individuals without cochlear dead zones presented 79.1% and 55.2%, respectively, indicating the benefit provided by amplifying the high frequencies. Under the same conditions mentioned above, patients with dead zones in their cochleas presented SRT 41.1% and 39.3%, respectively, without statistically significant difference between the two last values mentioned.

Vestergaard (2003)13 applied the TEN test and assessed speech recognition of a filtered speech in 22 individuals with moderate to profound sensorineural hearing loss, users of hearing aids, used to a substantial amount of sound amplification. The author’s goal was to check the TEN test feasibility and its capacity to reveal functional impairments. When the masking level was very close to the absolute threshold, many individuals had difficulties hearing because of noise. Nonetheless, this fact did not compromise the TEN test feasibility. Eleven individuals presented the possibility of having dead zones in their cochleas. The recognition of monosyllable words was carried out under four filtering conditions chosen individually with low-pass filters and another broad band condition, presented at 65 dB SPL, by means of a speaker, with individuals using their hearing aids. Individuals with cochlear dead zones presented a better performance than individuals without it for hard to hear speech, especially when hearing reduction happened because of the removal of high frequency tips. Patients with cochlear dead zones are used to perceiving filtered speech because their hearing works as a low pass filter. Individuals without these dead zones are more affected by removing the high frequencies from the hearing information. In some cases, there was no consistent information between the benefit of filtered speech and the spectral location of the dead zone. Other studies would be necessary in order to explain the results in a satisfactory way.

Gordo (2004)^14^ applied the TEN (HL) test in thirty adult individuals with bilateral descending sensorineural hearing loss. He used a two channel audiometer to control pure tone and noise separately, connected to a CD player. He investigated absolute hearing thresholds in 500, 750, 1000, 1500, 2000, 3000 and 4000 Hz in both ears, with TDH 49 phones and used 2 dB increments. In order to obtain TEN noise thresholds, he started at an intensity of 70 dB HL/ERB. When such level was insufficient to mask the absolute threshold, he gradually increased it to 85 dB HL/ERB, because he had an agreement with the patients that it would be the highest level used so as to not cause any discomfort. The sample was gathered in two groups: group 1- 15 individuals without cochlear dead zones; and group 2 with 15 individuals with cochlear dead zones in high frequencies. All the individuals underwent the investigation of the percentage index of speech recognition (PISR) and phrases recognition threshold, both carried out with and without competing noise. The author’s goal was to assess the relationship between hearing in the high frequencies and speech recognition in these patients, in order to establish differences in the benefits of the information obtained through the high frequencies sound amplification in a case-by-case basis. The speech tests were all proposed in different hearing conditions: without the hearing aid, with the hearing aid amplifying the frequency range between 100 and 8000 Hz (program 1) and with hearing aids with amplification in the range of frequencies restricted between 100 and 2560 Hz (program 2), avoiding gain in the high frequencies. Individuals without cochlear dead zones had a better speech test performance with and without noise, using the hearing aid provided in program 1. The group of individuals with cochlear dead zones had better results in all the tests with hearing aids in program 2. The author concluded that individuals without cochlear dead zones have more benefits from hearing aids amplifying the higher frequencies. In the presence of cochlear dead zones in the higher frequencies, individuals presented better performance with amplification in narrower frequencies range, that is, avoiding gain in the higher frequencies.

Almeida and Buzo (2005)15 investigated the presence of cochlear dead zones in a patient with sensorineural hearing loss and difficulty to accept bilateral amplification. They obtained absolute thresholds for air conduction for each ear in the frequencies of 250, 500, 1000, 1500, 2000, 3000, 4000, 5000, 6000, 8000 and 10000Hz first without and then in the presence of ipsilateral noise to the signal in the intensities of 30, 50 and 70 dB ERB. In the right ear, in which the patient has been using a hearing aid for 6 years now, absolute thresholds obtained with and without masking were similar. Now, for the left ear, the thresholds obtained in the presence of noise in the frequencies between 3000 and 10000 were higher, and this speaks for the presence of dead zones in this range of frequencies. The authors considered that the TEN test was an effective and simple procedure used to determine and outline the presence of dead zones in the left ear, matching patients’ complaints of the difficulties in accepting left ear amplification.

## DISCUSSION

We believe that, with the progress in diagnostic techniques in the area of clinical audiology, there came the possibility of better understanding the hearing of patients who are eligible to use hearing aids.

Thus, studies favoring cochlear dead zones diagnoses started having a growing importance for those who study and/or work with audiological diagnosis, as well as the selection and fitting of hearing aids.

Based on this concept of cochlear dead zones described by Moore and Glasberg (1997)^3^, studies were carried out in order to better fit patients who complain of hearing aid fitting.

Moore’s study (2001a)^4^ goes hand-in-hand with what is seen in the clinical practice, considering the difficulties patients have as far as sound amplification in given frequencies is concerned, and for that it is necessary to test these frequencies in order to check for the existence of dead zones in the cochlea.

In order to check for the existence of dead zones in the cochlea, Moore et al. (2000)^5^ developed the TEN, which is an efficient clinical test for the diagnosis of cochlear dead zones.

In the studies carried out by Baer, Moore and Kluk (2002)11, Vestergaard (2003)^12^, Gordo (2004)^13^ and Almeida and Buzo (2005)^14^, cochlear dead zones diagnosis was carried out through the TEN test, and the authors did not find difficulties to perform the test, and the results found were reliable and matched the theoretical assumptions related to the presence of cochlear dead zones. In the studies carried out by Baer, Moore and Kluk (2002)^11^, Vestergaard (2003)^12^ and Gordo (2004)^13^, there are references as to speech recognition, which is the main goal of the individual eligible to the use of hearing aids, and showed the importance of diagnosing dead zones in the cochlea: sound amplification is avoided in this region, and speech recognition improves.

Another diagnostic means is the psychophysical measure of tuning curves, described by Moore and Alcántara (2001)^8^ which we consider may be useful and reliable to identify cochlear dead zones and to outline the region involved. However, it bears the disadvantage of being a long and complex test, making it unfeasible for routine clinical use.

Eguti (2002)^9^ assessed cochlear dead zones using the technique proposed by Moore et al. (2000)^5^, however using the audiometer’s white noise. We believe the methodology proposed by the author is an excellent option for Brazil, both for research and for the clinical practice, for being more accessible and without raising costs for a reliable hearing diagnosis for those patients eligible for sound amplification.

We believe studies aimed at diagnosing cochlear dead zones must continue; and it is important that professionals involved in hearing rehabilitation include this assessment among routine audiological tests, when necessary.

## CONCLUSION

Audiograms provide important information so as to consider the presence of cochlear dead zones, however, this diagnosis may be carried out only after performing proper tests.

We also believe that the results from a patient eligible to use hearing aids depend, among other things, of our own knowledge regarding sound amplification necessary for each individual in particular, and the investigation of the presence or not of cochlear dead zones is one more information that may contribute to the success of hearing rehabilitation.
